# Collaboration between non-governmental organizations and public services in health – a qualitative case study from rural Ecuador

**DOI:** 10.3402/gha.v9.32237

**Published:** 2016-11-15

**Authors:** Olivia Biermann, Martin Eckhardt, Siw Carlfjord, Magnus Falk, Birger C. Forsberg

**Affiliations:** 1 Department of Public Health Sciences, Karolinska Institutet, Stockholm, Sweden; 2 Department of Medical and Health Sciences, Linköping University, Linköping, Sweden

**Keywords:** community participation, healthcare services, perception, primary healthcare, public private partnership

## Abstract

**Background:**

Non-governmental organizations (NGOs) have a key role in improving health in low- and middle-income countries. Their work needs to be synergistic, complementary to public services, and rooted in community mobilization and collective action. The study explores how an NGO and its health services are perceived by the population that it serves, and how it can contribute to reducing barriers to care.

**Design:**

A qualitative exploratory study was conducted in remote Ecuador, characterized by its widespread poverty and lack of official governance. An international NGO collaborated closely with the public services to deliver preventative and curative health services. Data were collected using focus group discussions and semistructured interviews with purposively sampled community members, healthcare personnel, and community health workers based on their links to the health services. Conventional qualitative content analysis was used, focusing on manifest content.

**Results:**

Emerging themes relate to the public private partnership (PPP), the NGO and its services, and community participation. The population perceives the NGO positively, linking it to healthcare improvements. Their priority is to get services, irrespective of the provider's structure. The presence of an NGO in the operation may contribute to unrealistic expectations of health services, affecting perceptions of the latter negatively.

**Conclusions:**

To avoid unrealistic expectations and dissatisfaction, and to increase and sustain the population's trust in the organization, an NGO should operate in a manner that is as integrated as possible within the existing structure. The NGO should work close to the population it serves, with services anchored in the community. PPP parties should develop a common platform with joint messages to the target population on the provider's structure, and regarding partners’ roles and responsibilities. Interaction between the population and the providers on service content and their expectations is key to positive outcomes of PPP operations.

## Introduction

Despite that the majority of the world's population is living in cities, the global rural population is now close to 3.4 billion people, with a concentration in low- and middle-income countries (LMICs) (1). In such settings, poverty is widespread and basic infrastructure is often deficient. The rural population faces specific health challenges, such as disease patterns, poor environmental conditions, and a lack of access to the health system. Although providing health services is an essential function of a country's health system, in many countries this function is still underdeveloped (2).

In many LMICs, the work of non-governmental organizations (NGOs) focuses on strengthening health services. Brown and Moore describe three core functions of health NGOs: delivering health services, providing capacity building, and influencing policy; each function is connected to different accountabilities (3). NGOs are able to increase the accessibility and quality of healthcare services for the rural population (4–6). They are typically close to the poor, have a flexible organization, encourage community participation, and have shown to be cost-effective (6–9). The actual number of NGOs that are active in the health sectors of LMICs is poorly documented, which may lead to a lack of recognition of their role at national and global levels (10). NGOs are often dependent on donors for financial support, arguably reducing their ability to articulate independent development approaches and skewing accountability to the donor, at the expense of the beneficiaries (6, 7, 11). The limitations of NGOs are most apparent in the resource constraints they face. These constraints can lead to inadequate service delivery through a lack of technical expertise in their staff, and poor resource mobilization (9). Therefore, in the design and implementation of NGO services, attention needs to be paid to sustainability (11).

On its own, the work of an NGO is not sustainable. For it to be considered sustainable, its work needs to be synergistic and complementary to public services (12) as well as rooted in community mobilization and collective action (13). A public private partnership (PPP) is therefore essential in NGO operations, with the NGO taking the role as the private actor. There is limited evidence in the literature on this form of PPP, and the related advantages and/or disadvantages in fulfilling the roles within the partnership.

Perceptions, irrespective of whether they represent reality, have real consequences for the acceptance of an NGO and its services (14–16). Perceptions have been described as difficult to measure (17): they are influenced by a person's education and culture; in turn, they influence his or her degree of health literacy, health-seeking behavior, and health status (18, 19). The evidence on the extent of this is, however, limited.

As is the case in many LMICs, Ecuador has a health system involving public, private for-profit, and nonprofit organizations, with limited institutional coordination (20, 21). NGOs are a part of Ecuador's private sector. The country has gone through years of political instability, with frequent changes in staff and in the leadership over time (21). In 2008, Ecuador approved a constitutional reform that aims for universal health coverage (22, 23). However, permanent access to health services, as stated in the 1998 constitution, is yet to be achieved (24).

## Methods

### Study setting

**Fig. 1:**
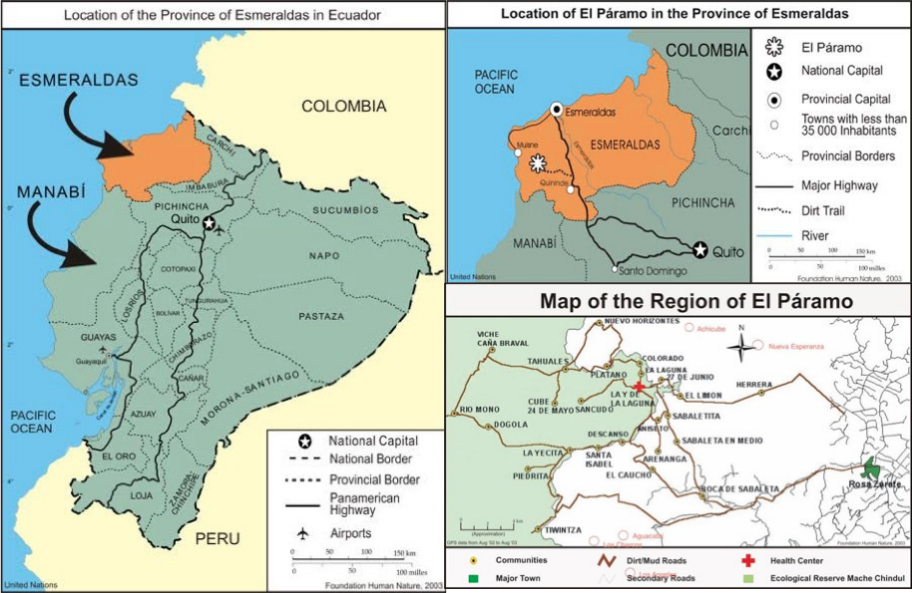
Map of El Páramo subdistrict catchment area and its location in Ecuador.

International volunteers founded the NGO ‘Foundation Human Nature’ (FHN) in 2001. A local voluntary health committee was formed, and it became the main body responsible for the construction of the health center. Both the local and the federal governments granted authorization for the establishment of the health center. The center is situated in La Y de la Laguna, the central village in a subdistrict known as ‘El Páramo’, Esmeraldas, in northwestern Ecuador (Fig. 1). Half of the health committee's board members were female, a prerequisite set by the international NGO. The health committee was officially registered as a local NGO with the Ministry of Social Affairs and was backed up with logistical and financial support from the international NGO, and with technical support from the local government. In 2002, this PPP between the local and international NGO, the local health committee, and the government commenced, after a long process of negotiations with the Ministry of Health (MoH).

The subdistrict El Páramo has around 5,000 inhabitants. Dirt roads during the rainy season impede access to healthcare, poverty is widespread, and official governance is lacking (19, 25). Alternative care providers to the health center in the central village are the public hospital and private providers in the district capital Rosa Zarate/Quinindé, or in other cities further away, traditional healers/birth attendants, local nonregulated pharmacies, and self-care (19). In the past, 29% of the population in El Páramo sought care in the health center during their most recent illness episode. This number is likely to have increased, particularly in light of the abolition of user-fees after the 2008 health reform (26).

Table 1 describes the respective responsibilities of the different actors in the PPP. Hereafter, the NGO refers to the Ecuadorian branch.

**Table 1:** PPP parties and their responsibilities

PPP party	Responsibilities
MoH	Recruiting, financing, and posting of national health professionals (who are mainly responsible for the daily services); providing materials and medicines
Local health committee	Owner of the health center and employer of local auxiliary and administrative staff; planning and management of annual activities and finances
Ecuadorian branch of NGO	Posting international volunteer doctors; sending health brigades to remote villages for medical and public health-related tasks; training voluntary CHWs from the regions’ villages
German branch of NGO	Recruiting international volunteer doctors; provision of financing to the local health committee; supporting the Ecuadorian branch of the NGO
International volunteer doctors	Clinical work in the health center, together with the national health professionals, or as their substitute in case of absence; participating in the health brigades; training of voluntary CHWs

PPP, public private partnership; MoH, Ministry of Health; NGO, non-governmental organization; CHW, community health worker.

A lack of knowledge regarding how an NGO and its health services are perceived by the population that it serves can be a hindrance to the delivery of health services. The aim of this study was to explore how an NGO and its health services are perceived by its target population and how it can contribute to reducing the barriers to care.

### Study design

An exploratory study design using qualitative research methods was chosen. Focus group discussions (FGDs) and semistructured interviews were adopted to gain an in-depth understanding of the informants’ perceptions. This approach can be useful when there is little knowledge about the subject of interest (27). FGDs and interviews were chosen as complementary methods (28): the FGDs were important to get a deep understanding of the population's perceptions, whereas key-informant interviews provided detailed insights from those that were more closely involved with the NGO. All participants in the study were from the geographical area that is served by the health center.

#### In-depth interviews

We purposively sampled and invited local key people, such as the leader of the local health committee, those in leadership positions during the inception phase of the health center, and the informal head of the region's central village. The health center's staff members and the director of the NGO were also invited. The health center's staff assisted in the recruitment, conducted through direct person-to-person communication. As an incentive, these participants received a free meal. Of the 16 participants that were invited to participate, 14 were interviewed: 6 were national staff members, 2 were expatriate staff members, and 6 were local key persons. Five of the participants were female. The nonparticipants were one national staff member and one local key person. Reported reasons for nonparticipation were lack of time and interest (Table 2).

**Table 2:** Characteristics of interview participants

Interviewee no.	Sex	Group	Comments
1	Male	Local key person	NGO director
2	Female	Local key person	Leader during initiation
3	Female	National staff member/health center	Cook and cleaner
4	Male	Local key person	Leader during initiation
5	Male	Local key person	Leader during initiation
6	Female	National staff member/health center	Midwife
7	Male	Local key person	Leader health committee
8	Male	National staff member/health center	Laboratory assistant
9	Male	National staff member/health center	Junior doctor
10	Female	National staff member/health center	Junior dentist
11	Male	Local key person	Former administrator
12	Male	Expatriate staff member/health center	Doctor
13	Female	Expatriate staff member/health center	Public health worker
14	Male	National staff member/health center	Public health worker

#### Focus group discussions

Participants were purposively sampled. Local community health workers (CHWs) were invited to participate when visiting the health center, or via a mobile phone, and community members were invited to participate when visiting the health center. Similarly to the in-depth interviews, the health center's staff and CHWs assisted in the recruitment, and participants were incentivized with a free meal. Of the approximately 55 people who were invited, 34 participated. Eleven of these participants were CHWs (eight males, three females), and 23 were community members (four women, seven men, six adolescent girls, and six adolescent boys). In total, six FGDs were carried out: group 1 with CHWs (three women, two men); group 2 with CHWs (six men); group 3 with community members (seven men); group 4 with community members (five women); group 5 with community members (seven adolescent girls); and group 6, also with community members (seven adolescent boys). The majority of the nonparticipants were community members whose reasons for nonparticipation were lack of time and interest (Table 3).

**Table 3:** Characteristics of interview and FGD participants

FGD no.	Constellation	FGD group	Observations
1	Three women, two men	CHWs	Two participants (women) from villages distant to the health center (ca. 6–7 h walking distance), three participants from villages closer to the health center (ca. 1–2 h walking distance), three invitees abstained due to lack of time
2	Six men	CHWs	Three participants from villages distant to the health center (ca. 4–5 h walking distance), two participants from villages closer to the health center (ca. 1–2 h walking distance), one participant from La Y de la Laguna, two invitee abstained due to lack of time
3	Seven men	Community members	Three participants from villages distant to the health center (ca. 4–5 h walking distance), four participants from La Y de la Laguna, one invitee abstained due to lack of time, three invitees abstained due to lack of interest
4	Five women	Community members	Two participants from villages distant to the health center (ca. 3–4 h walking distance), three participants from villages closer to the health center (ca. 1–2 h walking distance), four invitees abstained due to lack of time, one invitee abstained due to lack of interest
5	Six girls	Adolescents	Five participants from villages closer to the health center (ca. 1–2 h walking distance), one participant from La Y de la Laguna, two invitees abstained due to lack of time, one invitee abstained due to lack of interest
6	Six boys	Adolescents	One participant from village distant to the health center (ca. 6–7 h walking distance), six participants from villages closer to the health center (ca. 1–2 h walking distance), four invitees abstained due to lack of time

FGD, focus group discussion; CHW, community health worker.

### Data collection

The interviews and FGDs were based on interview guides that were developed by the authors (Table 4). Only minor adjustments were made after two pilot interviews; thus, the pilot interviews were included in the study (29–31).

**Table 4:** Question guides for FGDs and key-informant interviews

Question guide for FGDs
1. Let us start with presentations of yourselves:
a. What is your name?
b. Where do you live?
c. How much time did you need to reach La Y de la Laguna [the central village where the health center is located]?
2. Please think about a sick person in your community. Let us collect some thoughts: How has healthcare for sick people changed in the community until today?
3. Looking back, do you remember what your first impression of a health brigade was?
4. Nowadays, how would you explain the NGO FHN and the health brigade to a friend?
5. Now we will talk about the satisfaction with the brigades and different people who work in the healthcare center:
a. Could you please describe what you most appreciate?
b. Could you please describe what you dislike?
6. Could you please complete the phrase: ‘If I was working with the NGO FHN or the health brigades, I would change one thing …’
7. Could you imagine getting involved in the NGO FHN or the health brigades?
a. How would you like to collaborate?
8. Do you have any other comments?
Question guide for key-informant interviews
1. Could you say a few words about yourself?
2. How can the development of the NGO FHN be briefly described?
3. What do you think of how the role of the NGO FHN – the organization and the brigades – is perceived by the population in El Páramo?
4. Do you think that people are satisfied with the NGO FHN?
5. Do you think there is a difference between the perception of medical doctors from Ecuador and foreign medical doctors?
6. What do you think about the participation of the community in the organization?
7. How can participation of the community be realized?
8. If you could change one thing, what would it be?

All of the interviews and FGDs were audio recorded. A research assistant, present during the FGDs, transcribed each interview and FGD *ad verbatim* after it took place. The recordings and transcriptions were double-checked by the main author to increase credibility. Participants were informed of the academic goal of the study, before the interview or FGD took place.

Fourteen face-to-face key-informant interviews were conducted. The interviews were carried out in private rooms located in the health center, or in participant's homes, and lasted an average of 30 min. The six FGDs were conducted in private rooms also located in the health center, and they lasted from 30 to 60 min. All FGDs and interviews were conducted in Spanish.

### Data analysis

Data were collected and analyzed until a point at which no emergent patterns were found (31); that is, new interviews continued to be planned until saturation was reached. Transcripts were read multiple times by OB and ME. The analysis was based on the total database, and the coding process was done manually.

All data were analyzed using conventional content analysis. This analysis is described by Graneheim and Lundman (32) as when meaning units are identified and condensed, then abstracted inductively to create codes. We subsequently combined these codes into subcategories and finally, categories, based on the manifest content of the transcripts. OB and ME identified themes independently and discussed these themes until consensus was reached. SC validated the findings and checked the results of the analysis to enhance credibility. Member checking took place with 10 participants, who had taken part in FGDs and interviews, enhancing the credibility of the analysis and interpretation of the results. A description of the coding tree was not shared with participants during these checks. In the presentation of the results, selected quotations are presented to reflect common answers from the respondents, and differences in responses are also highlighted.

Perceptions of the NGO and its services are portrayed in various ways. The following themes were identified:

1) PPP (categories: services, strengths and weaknesses);

2) NGO and its services (categories: volunteer doctors, health brigades); and

3) Community participation (categories: development, CHWs, participation on the individual level).

### Ethics

Ethical approval was obtained from the bioethics committee at the Pontificia Universidad Católica del Ecuador (no. Oficio-CBE-001-2013). Written approval was granted by the Ecuadorian NGO FHN and the local health committee for the El Páramo region.

Written informed consent was obtained from the participants before the start of each key-informant interview and FGD. In the case of illiteracy, verbal informed consent was obtained. The anonymity and confidentiality of the participants were ensured by removing all identifiers except the respondent category in the presentation of the results. Best practice guidelines for qualitative research (33) were applied to increase the trustworthiness of this study (34).

## Results

### Public private partnership

#### Services

Participants in interviews and FGDs frequently expressed their gratitude for the health center being available in their region, and for providing services.

We are calm because the health center exists. (interviewee 4)

In the FGD with women from the community, the provision of medication was welcomed. However, some participants experienced a lack of medication.

A: I would tell my friend to come here [to the health center], that there are medications.

B (interrupts): But sometimes still, medications are missing. They should provide a bit more because sometimes medications are lacking. Simply, sometimes, there are no medications for family planning because sometimes I have come and there were none [medications for family planning] […].

Interviewer (looking to the other participants): Do you have similar experiences?

C (shrugs shoulders): I don't, but many people say that they do.

D: Me neither, here [at the health center] I find the medications.

Participants from an FGD with CHWs expected support from the NGO to the health center to continue in the future through financing, the provision of infrastructure, and tools.

Participants in FGDs and interviews spoke of the attentiveness and availability of the medical staff. Despite the appreciation of ‘warmth and quality of the attention’ (interviewee 6) and the feeling of their needs being satisfied, participants in FGDs made different critical points that were mostly related to the availability of medical staff. In one of the FGDs with CHWs, the wish of having a permanent medical doctor was discussed.

A: [It would be good] if there was a doctor permanently. Sometimes one, or some person from the community, could suffer from something serious, and being able to go the health center and be sure that the doctor will be there […].

B (nods): The most desirable would be to have a permanent doctor because sometimes I feel fine and healthy, and after a little while this can change […]. Once, a woman, after some four, five years of good health, changed, and she almost died. And like around 10 pm, there was no doctor here [at the health center] to attend [her].

#### Strengths and weaknesses

The collaboration between the three parties that form the PPP was mentioned by interviewees: naming advantages (benefits through a contract with the MoH, continued collaboration despite controversies), and disadvantages (difficulties due to each parties’ own interests, distrust between organizations). One interviewee mentioned personal conflicts between the leaders of the NGO and the local health committee, whereas another spoke with frustration at the desire ‘that the Directors sit down together to share ideas and continue working together’ (interviewee 3).

In interviews, unclear roles and responsibilities in the PPP were stated as a weakness.

Few persons from the communities understand the roles. (interviewee 3)

[There is a need to] explain the situation with the three parties. (interviewee 7)

Interviewees conveyed the need to generate local capacity, and for local leadership, with leadership perceived as strong in the early days of the project. Interviewees also talked about the NGO's goal to be a sustainable health center that would no longer need NGO support.

In an FGD with CHWs, communication between the health center staff and community members was discussed: CHWs perceived that it was not the lack of communication and information that led to people's decisions not to visit the health center, but misbeliefs and rumors.

A: It's very evident, the lack of communication and information of the people with the health center. That's why there are these types of rumors among people.

B: But it seems that this [lack of communication] is an excuse […].

C (nods): Despite having information… if it's not their individualism.

D (nods): That is true because today in the morning, the people from my community […] there were only four people who came.

E (raises voice): To be honest, if we talk about a percentage […] it's only 10% of the people who really don't know about the services of the health center. […] I have met people who tell me that they don't come because they [staff at the health center] are charging [a fee]. Or there are people that, once there is a meeting of people in the community announced, they don't go, you never see them. These people are, sometimes, those that don't know about the services that are offered there [at the health center]. […] Well, this ignorance is why the people don't go [to the health center] […].

#### NGO and its services

##### Volunteer doctors

Overall, participants in interviews and FGDs brought up trust issues. Some interviewees had more trust in the volunteer doctors. However, one interviewee had the perception that ‘foreigners come to take advantage of the people in “El Páramo” instead of helping them’ (interviewee 5).

Many appreciated the support given by the volunteer doctors and valued the permanent presence of volunteers. Furthermore, they were described as providing better treatment, being better prepared, having a better education, and being more professional. An interviewee described the volunteers as ‘totally dedicated and helping the patients – no matter which time of the day’ (interviewee 1). In different FGDs, volunteers were mostly perceived as having a good attitude, being friendly, and patient.

Different interviewees named insufficient Spanish skills as a weakness of volunteer doctors. During one FGD, an adolescent girl said: ‘We don't understand them, they don't understand us’ (focus group with girls). FGD participants spoke of the language barrier, but highlighted that they received good care in spite of this.

Interview participants also mentioned a lack of communication skills and insufficient knowledge of local diseases and the local health system as weaknesses.

One FGD participant drew the attention away from positive and negative perceptions of doctors, emphasizing the collaboration between foreign and Ecuadorian doctors, which he perceived as beneficial.

I don't care about the issues that people have. It's about having doctors here that attend patients. […] The majority thinks that it's very good that they [the expatriate doctors] work together with Ecuadorian doctors – in this way they are increasing the learning here. (focus group with men)

##### Health brigades

The appreciation of the support given by health brigades was frequently mentioned, considering the brigades traveling far in challenging circumstances to provide healthcare. Brigades were perceived as important because of economic support (community members save time and money by not traveling to the health center) and for receiving a health check-up.

The brigades are grandiose: They are coming to see patients in their own houses (focus group with CHWs). And: When I got an examination, I noticed that I had parasites. (focus group with CHWs)

Participants perceived the brigades as being particularly important for accessing health for the population living far away, and for women and children. Some individuals did not want to leave their villages and were therefore grateful for the brigades for providing outreach services. Others perceived women and children in particular to benefit, due to ‘machismo’, whereby they were not allowed to leave their homes.

It is very important because […] machismo still exists. So, the wives and children don't leave [the village] because it's like this. So, it's good that the brigades come to help them. (interviewee 2)

An interviewee perceived the brigades as being ‘incomplete’ because of their failure to carry sufficient medication. The perceived lack of medication, including vaccines, was also discussed in an FGD. In some cases, people were sent to the district capital Quinindé when medication was not available.

Participants perceived the implementation of public health measures as one of the big opportunities for the health brigades. ‘They [the brigades] have shown results: we have fought against malaria’ (interviewee 4). The brigades were also perceived to enhance the motivation of individuals to engage in health seeking behavior.

### Community participation

#### Developments

Participants in interviews and FGDs described active community support in the inception phase. There was ‘collaboration from everybody to construct the health center’ (interviewee 5). Interviewees talked about the subsequent dependence of the NGO that they perceived as leading to ‘incapacity of the people’ (interviewee 1). Community support was described as being necessary for the sustainability of the project.

#### Community health workers

Overall, the network of CHWs and the capacity-building workshops given for the CHWs at the health center were cherished. ‘In this way, we move forward’ (focus group with boys).

One interviewee perceived the biggest weakness as the lack of interaction and communication of community members with CHWs. In one FGD with CHWs, a participant described the challenges of interacting with fellow community members.

They [community members] only help you when the brigade comes because the brigade attends them. When there is a brigade, the people say ‘yes, yes’, but once the brigade leaves and only the CHW is there, no […]. They [community members] say that they [CHWs] don't know […]. (FGD with CHWs)

Participants perceived the need for local leadership of the CHWs and for community members who were interested in becoming a CHW. Several interviewees perceived incentives as necessary for CHWs to maintain their network.

Participants also pointed out a misconception that CHWs earned a salary, which was said to have led to community members’ increased expectations of CHWs’ performance.

One topic that came up repeatedly in FGDs and interviews was capacity building for, and by, the CHWs. Besides public health–related work that CHWs were expected to learn about, their capacity building was seen as ‘necessary for the medical attention’ (interviewee 5) and to learn ‘going with the doctor to attend patients’ during health brigades (*focus group with men*). Participants in FGDs also commented on the interaction with the community as an opportunity for the CHWs: CHWs were perceived to both bring the community together and involve them.

#### Community participation on the individual level

Interviewees perceived individualism as an obstacle to community participation.

It is difficult to initiate effective participatory processes. The people are very individualistic. (interviewee 1)

Furthermore, an interviewee perceived community members as being passive: ‘Sometimes people think that everything will be served to them’ (interviewee 5).

An interviewee stated that the CHWs contributed to the development of the health center. Participants perceived that CHWs motivated other community members to participate in ‘mingas’ (the gathering of community members to jointly do work) and that the CHWs organized different kinds of meetings and activities related to health in their communities. There were no substantial differences found between the information collected from the FGDs and interviews. Participants expressed their gratefulness for the positive development in health in El Páramo since the health center had been set up.

## Discussion

The NGO is perceived positively because the community members frequently associate it with healthcare improvements. These perceptions are likely to have a positive influence on the acceptance of the NGO and its services (14–16). The association between the experience of individuals with health services and the structure of the health services provider tends to be unclear. The operational integration of NGOs and government providers can lead to difficulties in distinguishing between the different actors (4). Conflicts related to (perceived) accountability can consequently arise (2), for example, when individuals perceive the NGO responsible for primary care as also being accountable for providing highly specialized medical services.

Uncertainty about the providers’ roles and responsibilities among community members leads to misunderstandings and hence dissatisfaction with the NGO and its services. For example, participants attach blame to the NGO for the absence of medical staff at the health center, which is in fact, not the responsibility of the NGO (who are responsible for the volunteer doctors only), but of the MoH (who provide local staff with set working hours).

In many cases, community members do not seem to care who, or what, the NGO is, nor what its actual tasks are. Participants rarely distinguish between the roles of the three PPP parties. The most important factor seems to be that they are provided with care at all. However, Donini et al. (16) found that certainty about the different roles and responsibilities within the PPP can lead to reasonable expectations, understanding of the situation and thus enable constructive criticism. A better understanding of the provider's structure could enhance the population's trust in the organizations and, in turn, give the organizations a stronger sense of responsibility for their community (15).

Perceptions have been described as difficult to weigh. There can be confusion regarding the population's perceptions and their satisfaction, and it can be difficult to determine whether variations in perceptions can be attributed to differences between expectations and actual experiences (17).

The population perceives the PPP as one provider, and not the NGO individually (as previously described by Gilson et al.) (5). Thus, the individual parties of the PPP have a joint responsibility to increase the population's understanding of the provider's structure, including roles and responsibilities within the PPP (17, 35).

When talking about the NGO and its services, participants most commonly referred to the services by the volunteer doctors and the health brigades, both of which contribute to increasing access to healthcare at the grassroots level. The extent to which NGOs provide health services in LMICs was also mentioned in multiple studies, attributing this to the improvement in the population's access to care (2, 3, 12).

Participants’ perceptions of the health center's doctors are complex, because they involve challenges around trust. A lack of trust in Ecuadorian doctors is related to their often young age, or doubts in the quality of their education. Mistrust in foreign doctors is linked to a lack of knowledge of the local context and/or language barriers. Trust and acceptance for an organization are achieved by individuals working within the organization gaining trust (15). To overcome these trust issues, and to enhance teamwork and communication, participants emphasized the need for collaboration between Ecuadorian and foreign doctors. Both parties would benefit from mutual involvement, through skill and knowledge sharing, which has similarly been described in the literature (11, 35).

The importance of health brigades that can reach peripheral communities emerged in this study. Health services need to be close to the population they are serving; this need for proximity is further emphasized in humanitarian work. Studies have shown that despite the presence of the humanitarian aid organization *Médecins Sans Frontières* (*Doctors Without Borders*), remote villages were still not reached with healthcare services (15). For some women and their children, the health brigades make healthcare accessible, in part, because their husbands do not allow them to leave their villages. These women and their children may also benefit in other ways from the contact with national and international health workers. A study in Nicaragua found empowerment of local women as a (initially unintended) positive side effect of a similar project (36). Some of the CHWs that are working with the NGO are female: contact between them and local women during health brigades may have a positive effect on the latter.

There are misconceptions concerning the CHWs (such as CHWs earning a salary), making collaboration with community members challenging. Collaboration is further challenged by community members’ individualism and lack of leadership. It has been described as challenging to bring the notion of sustainability closer to the population and to stimulate them in being proactive in their support of the idea of sustainable health service delivery (2). Local leadership is desired, and efforts should be made to strengthen the network of CHWs.

To sustain the network of CHWs and to increase their capacity to take up leadership roles, CHWs should be incentivized. The involvement of community members in the work of the NGO seems to have declined and might have led to a diminishing feeling of ownership and a failed communication flow, ultimately leading to perceptions not presenting reality. When the NGO started, it had a strong focus on service delivery. With developments, its focus broadened and activities increasingly included capacity building. Brown and Moore (3) describe challenges for NGOs that start off as service delivery organizations, but subsequently move toward, and make the shift to, a capacity building function. These challenges arise because the population's understanding and expectations of the NGO may not shift accordingly and thus lead to unrealistic expectations and dissatisfaction.

Even though the health center in the central village brought health services closer to the population in the region; brigades and CHWs that reach people in their communities were regarded as of utmost importance. They bridge the physical and the communication-related gap between the population and its health services, with the latter potentially even more powerful in creating a barrier to the population accessing these health services.

Further research should be conducted to understand the possibilities of engaging community members in health service development, linking NGO work and public services, communication within a PPP and with a target population, as well as a target population's perceptions of providers and services.

This study had some limitations. The researcher who collected the data has a foreign background and was not a native Spanish speaker, potentially causing bias due to language and cultural differences. We conducted member checking of preliminary results to reduce possible bias and to enhance the credibility and dependability of this study (33).

We acknowledge that this study was conducted in one subdistrict in rural Ecuador. Consequently, generalizability of findings may be limited because health system gaps are, to a large extent, context specific. Nevertheless, we trust that the lessons from these results are transferable to other rural districts in Ecuador and, at least, partially transferable to other rural settings with similar health system characteristics. This study may be of value in the planning and design of further studies.

## Conclusions

The marginalized population that the NGO is serving has generally positive perceptions of the NGO, because it associates the NGO with healthcare improvements. The lack of clarity regarding the association between experiences of health service delivery and the PPP provider structure have contributed to misunderstandings, and subsequent dissatisfaction, with the organization and its services. Although rarely differentiating who or what the NGO is, and what its actual tasks are, it seems most important to the population to be provided with care at all.

To avoid unrealistic expectations and dissatisfaction, and to increase and sustain the population's trust in the organization, an NGO should operate in a manner that is as integrated as possible in existing structures. Their services should be anchored in the community and conducted closely to the target population. PPP parties should develop a common platform with joint messages to the target population on the provider's structure, and on the respective partners’ roles and responsibilities. The interaction between the population and the partners on service content and their expectations is key to ensuring positive outcomes of PPP operations.

## Authors’ information

OB has a Bachelor of Science (BSc) in Health Communication and a Master of Science (MSc) in Global Health Sciences. ME is a Medical Doctor (MD), MSc in International Health, and Doctor of Philosophy (PhD) student. SC is a Registered Physiotherapist, Master of Public Health and PhD in Public Health. MF is MD, PhD and Associate Professor. BF is a BSc in Economics, MD, MSc in Public Health, PhD and Associate Professor in Global Health.

## Paper context

The work of non-governmental organizations and public services need to be synergistic and rooted in collective action to successfully deliver health services. Non-governmental organizations have to operate at the implementation level, close to the population they serve, to be effective. Partners in a public private partnership should inform their target population about the provider's structure to increase the population's understanding, monitor perceptions, and communicate the non-governmental organization's functions to avoid unrealistic expectations, dissatisfaction, and mistrust.
